# Genetic Regulation of α-Synuclein mRNA Expression in Various Human Brain Tissues

**DOI:** 10.1371/journal.pone.0007480

**Published:** 2009-10-16

**Authors:** Colton Linnertz, Laura Saucier, Dongliang Ge, Kenneth D. Cronin, James R. Burke, Jeffrey N. Browndyke, Christine M. Hulette, Kathleen A. Welsh-Bohmer, Ornit Chiba-Falek

**Affiliations:** 1 Institute for Genome Sciences & Policy, Duke University Medical Center, Durham, North Carolina, United States of America; 2 Division of Neurology, Department of Medicine, Duke University Medical Center, Durham, North Carolina, United States of America; 3 Joseph and Kathleen Bryan Alzheimer's Disease Research Center, Duke University Medical Center, Durham, North Carolina, United States of America; National Institutes of Health, United States of America

## Abstract

Genetic variability across the *SNCA* locus has been repeatedly associated with susceptibility to sporadic Parkinson's disease (PD). Accumulated evidence emphasizes the importance of *SNCA* dosage and expression levels in PD pathogenesis. However whether genetic variability in the *SNCA* gene modulates the risk to develop sporadic PD via regulation of *SNCA* expression remained elusive. We studied the effect of PD risk-associated variants at *SNCA* 5′ and 3′regions on *SNCA*-mRNA levels *in vivo* in 228 human brain samples from three structures differentially vulnerable to PD pathology (substantia-nigra, temporal- and frontal-cortex) obtained from 144 neurologically normal cadavers. The extensively characterized PD-associated promoter polymorphism, Rep1, had an effect on *SNCA*-mRNA levels. Homozygous genotype of the ‘protective’, Rep1-259 bp allele, was associated with lower levels of *SNCA*-mRNA relative to individuals that carried at least one copy of the PD-risk associated alleles, amounting to an average decrease of ∼40% and >50% in temporal-cortex and substantia-nigra, respectively. Furthermore, SNPs tagging the *SNCA* 3′-untranslated-region also showed effects on *SNCA*-mRNA levels in both the temporal-cortex and the substantia-nigra, although, in contrast to Rep1, the ‘decreased-risk’ alleles were correlated with increased *SNCA*-mRNA levels. Similar to Rep1 findings, no difference in *SNCA-*mRNA level was seen with different *SNCA* 3′SNP alleles in the frontal-cortex, indicating there is brain-region specificity of the genetic regulation of *SNCA* expression. We provide evidence for functional consequences of PD-associated *SNCA* gene variants in disease relevant brain tissues, suggesting that genetic regulation of *SNCA* expression plays an important role in the development of the disease.

## Introduction

Alpha-synuclein (*SNCA*) (Ensembl: ENSG00000145335; OMIM, Online Mendelian Inheritance in Man: MIM 163890) was the first gene found to be involved in Parkinson's disease (PD[MIM 168600]). SNCA aggregates have been identified within Lewy bodies, the pathological hallmark of PD [1]. Also, mutations [2]–[4] and copy number variations [5]–[9] in the *SNCA* gene have been identified in a few families with an early onset, autosomal dominant form of PD. Furthermore, accumulated evidence suggests that elevated levels of wild type SNCA lead to neuronal dysfunction and are sufficient to cause early onset familial PD. Genomic triplication of the region containing *SNCA* was shown to result in four fully functional copies of *SNCA* and 2-fold over-expression of *SNCA* mRNA and protein and a highly penetrant early-onset PD phenotype with cognitive impairment and autonomic dysfunction [10], [11]. Similarly, duplications of the wild-type *SNCA* gene result in a 1.5-fold elevation of *SNCA* expression and a slightly later onset of heritable PD that is characterized by a lower penetrance rate and a ‘milder’ phenotype than for the triplication [6]–[9], demonstrating the dose-dependent effect of SNCA on disease presentation. Furthermore, elevated levels of *SNCA*-mRNA have been reported in midbrain tissues [12] and in individual substantia nigra dopaminergic neurons from sporadic PD *post mortem* brains compared to controls[13]. These observations emphasize the importance of *SNCA* dosage and expression levels in PD pathogenesis.

Several association studies have demonstrated that genetic variability across the *SNCA* locus is associated with susceptibility to sporadic PD [14]–[18]. Based on HapMap data *SNCA* has two major linkage disequilibrium (LD) blocks, a 5′ block that extends to the promoter-enhancer region and a 3′ block that comprises the 3′untranslated-region (UTR) and the 3′ region of the gene [15], [18]. These studies confirmed the association of variants within both *SNCA* 5′ and 3′ LD-blocks with PD-risk, suggesting that the genetic regulation of *SNCA* expression might be mediated through different molecular mechanisms (transcriptional and post transcriptional) and could have an important role in the development of the disease. Previously, we extensively characterized the best confirmed associated genetic variation, Rep1, a polymorphic nucleotide repeat site located ∼10 kb upstream of the *SNCA* transcription start site [19], [20]. Using a reporter assay in a transiently transfected neuronal cell line [21], [22] and a transgenic mouse model [23], we demonstrated that *SNCA*-Rep1 had a reproducible effect on regulatin*g* transcriptional activity. In both model systems, the extended risk allele showed increased expression of the reporter construct and the human transgene, respectively; while the shorter PD-‘protective’ allele was associated with lower expression levels [21], [22].

Here we aim to reveal the functional consequence of genetic variations in the *SNCA* genomic region. We studied the effect of the Rep1 variant as well as other PD risk-associated variants on *SNCA*-mRNA steady state levels *in vivo* in three human brain structures differentially vulnerable to PD; i.e. frontal cortex, temporal cortex and mid-brain including the substantia nigra (SN). Our comprehensive analysis was performed using *post mortem* matched brain tissues from unaffected individuals to directly assess the genetic contribution to the regulation of *SNCA* expression, avoiding other confounding factors arising from the neurodegeneration associated with PD.

## Results

### Effect of secondary (non-genetic) variables on SNCA-mRNA level

*SNCA*-mRNA fold levels (*SNCA/SYP*) were measured in 228 brain tissue samples obtained from 144 subjects (83% white, 56.5% males; Table 1). First, we assessed the correlation of SNCA-mRNA expression with confounding factors that might affect RNA levels. All midbrain including substantia nigra (SN) samples were obtained from white individuals. *SNCA* mRNA folds levels in midbrain including SN (n = 34) were not correlated with sex (P = 0.187), age (P = 0.735), or PMI (p = 0.177). Similarly, no correlations of SNCA-mRNA levels were observed in temporal (n = 77) and frontal cortex (n = 117) with sex (P = 0.49, 0.46), race (P = 0.59, 0.14), age (P = 0.35, 0.742), or PMI (P = 0.85, 0.7).

**Table 1:** Demographic description of the brain samples.

	Subjects	Tissues	FC	TC	SN	Subjects with multiple tissues	FC+TC+SN	FC+TC	FC+SN
**Total no.**	144	228	117	77	34	77	7	68	2
**White (%)**	83.0		81.2	72.7	100		100	72.1	100
**Male (%)**	56.5		53.5	51.4	53.6		0	50.0	0
**Age at Death** (mean±S.E.M)	65.1±18.1		69.3±16.9	69.0±17.6	55.6±18.1		82.4±5.8	69.8±17.2	81.5±4.5
**PMI** (mean±S.E.M)	15.2±6.8		15.4±7.5	13.3±7.0	13.0±4.2		7.5±4.9	13.3±7.1	14±2.0

FC- frontal cortex, TC-temporal cortex, SN-substantia nigra. PMI- post mortem interval.

**Figure 1:**
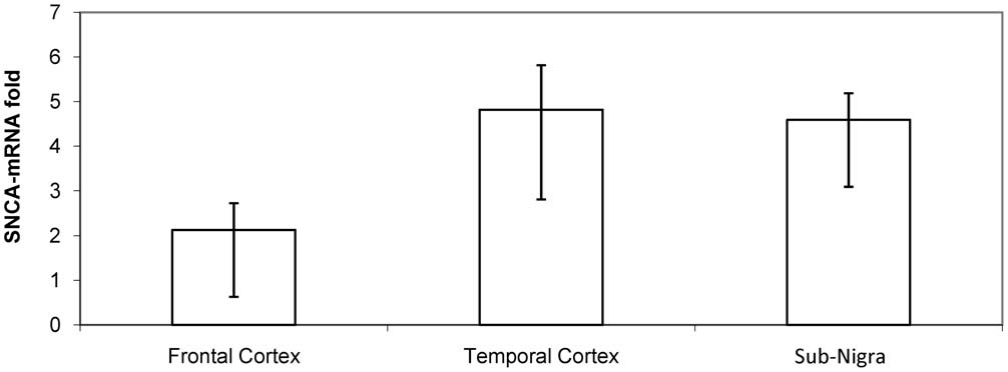
Average fold expression of *SNCA*-mRNA in three matched brain regions obtained from the same subjects (n = 7). Fold levels of *SNCA*-mRNA were assayed by real-time RT-PCR using TaqMan technology and calculated relative to the geometric mean of *SYP- and ENO2-* mRNAs reference control using the 2^−ΔCt^ method. The bar graph presents the average of *SNCA*-mRNA fold expression (mean±S.E.M) of the 7 subjects for each brain region. SN-substantia nigra; TC-temporal cortex; FC- frontal cortex.

Next, the effect of specificity of the brain tissue region on SNCA expression was assessed. To carry out this analysis we used matched samples, i.e. samples of different brain structures obtained from the same cadaver. Frontal cortex showed significantly lower levels of SNCA-mRNA compared with midbrain including SN (n = 9; P = 0.001) and temporal cortex (n = 75; P = 1.6×10^−9^). The average SNCA-mRNA fold levels observed in the frontal cortex samples was approximately 50% less from the average SNCA-mRNA fold levels detected in the temporal cortex and in the midbrain including SN of the same individuals. Comparison of *SNCA* mRNA fold levels between matched samples of temporal cortex and midbrain including SN revealed no differences (n = 7, P = 0.37). Furthermore, we were able to carry out a direct comparison of all three brain regions in 7 cadavers from whom all brain regions were available, showing that the average fold expression levels of *SNCA*-mRNA are similar in midbrain including SN and temporal cortex and nearly twice the average fold levels observed in the frontal cortex (Figure 1). Thus, temporal cortex may serve as a mirror for *SNCA*-mRNA expression levels in the substantia nigra.

### Effect of variants in the 5′ region of *SNCA* on SNCA-mRNA levels in different brain tissues

**Figure 2:**
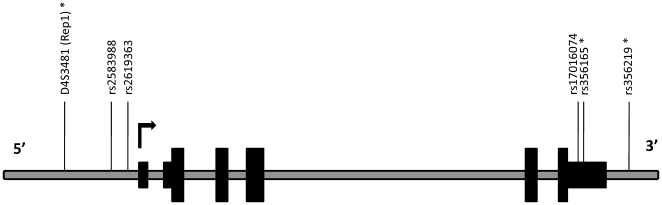
A schematic representation of the human SNCA gene with the relative positions of the markers. Organization of the human *SNCA* locus: translated exons, wide black solid boxes; 5′ and 3′UTR, narrow black solid boxes; introns and intergenic regions, grey line. The relative positions of the genetic variants are indicated above, asterisks designate variants that were associated with *SNCA*-mRNA.

We studied the effect of variants in the promoter enhancer region of *SNCA* on *SNCA* mRNA levels. Three polymorphisms were tested: two *SNCA* 5′ region tagging SNPs (rs2619363 and rs2583988) and Rep1 (Figure 2).

All samples were genotyped for both the rs2619363 and rs2583988 SNPs; Table 2 summarizes the allele frequencies in our samples. The tagging SNPs rs2619363 and rs2583988 in the promoter of the *SNCA* gene did not show any correlation to *SNCA* mRNA in the midbrain including SN (P = 0.58, 0.92) the temporal cortex, (P = 0.68, 0.95) or the frontal cortex (P = 0.68, 0.84).

**Table 2:** Rep1 allele frequencies of the study group.

Rep1	All No.(%)	Caucasians No.(%)	Maraganore *et al*. (JAMA, 2007) No.(%)
***259***	88(0.31)	71(0.304)	1413(0.27)
***261***	172(0.606)	151(0.645)	3579(0.68)
***263***	23(0.08)	12(0.051)	312(0.06)
***265***	1(0.004)	0	0

No.- total number of alleles (2 per individual); % allele frequency.

**Figure 3:**
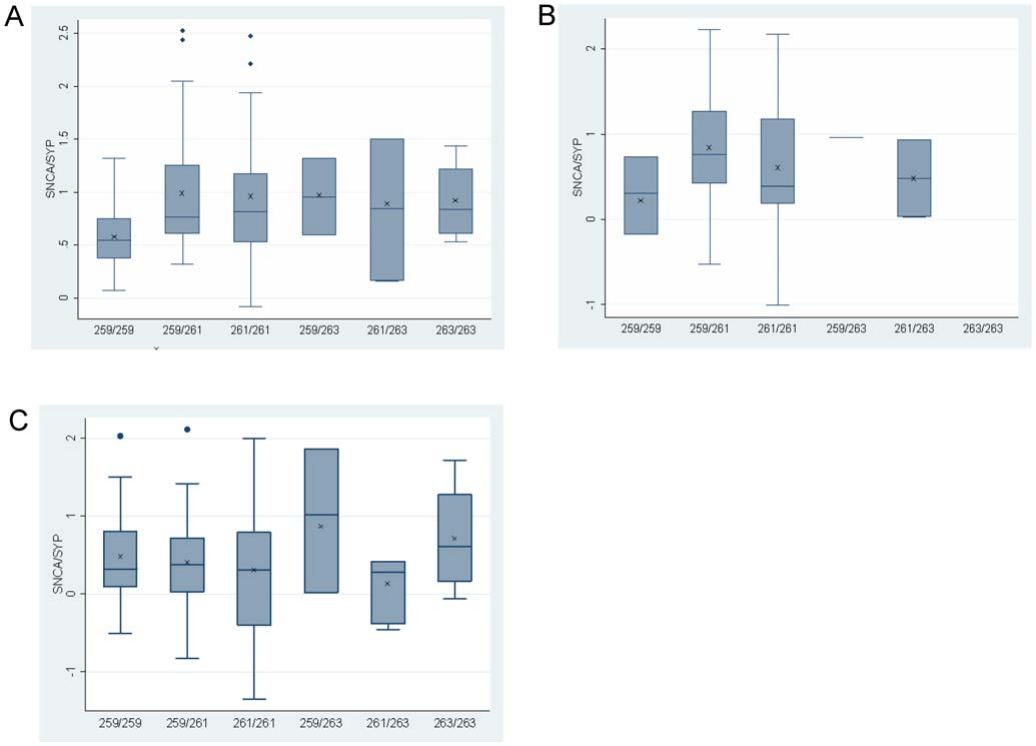
Effect of the *SNCA*-Rep1 promoter genotypes on human *SNCA*-mRNA expression levels in human brains. Individuals were genotyped for Rep1. Three brain regions were analyzed: temporal cortex (A), midbrain including SN (B) and frontal cortex (C). In each brain region fold levels of human *SNCA*-mRNA were assayed by real-time RT-PCR using TaqMan technology and calculated relative to human *SYP*-mRNA reference control using the 2^−ΔCt^ method. (A) Analysis of the temporal cortex showed that the protective genotype 259/259 correlates with lower *SNCA*-mRNA levels then the five other genotypes (P = 0.02). The association trend was confirmed in a subset of samples using also the *GAPDH* and *ENO2* reference genes (Table S2). (B) In the midbrain including SN the 259/259 correlates, with lower *SNCA*-mRNA levels. (C) No correlations of Rep1 genotypes with *SNCA*-mRNA levels were detected in the frontal cortex. For each genotype the box plot represents the analysis performed using all brain samples available from the specific brain region, each of which was analyzed twice independently, each time in duplicate. The average values are presented in ‘X’. The box plot shows the median (horizontal line inside the box) and the 25^th^ and 75^th^ percentiles (horizontal borders of the box). The range between the 25th and 75th percentiles is the interquartile-range (IQR). The whiskers show the minimal and maximal values inside the main data body.

We then tested for correlation with Rep1 genotypes. A summary of Rep1 allele frequencies of the studied samples is presented in Table 3. Analysis of the temporal cortex (n = 77) indicated that individuals homozygous for the PD-‘protective’ genotype 259/259 (n = 8) had lower SNCA-mRNA levels than individuals carrying the 259/261, 261/261, 259/263, 261/263 and 263/263 genotypes (Figure 3A; P = 0.02). In the temporal cortex Rep1 259/259 demonstrated an average 0.59 fold *SNCA*-mRNA expression level compared with an average 1.00, 0.94, 0.95, 0.83 and 0.90 fold expression level of *SNCA*-mRNA in the five other genotypes 259/261, 261/261, 259/263, 261/263 and 263/263 genotypes carriers, respectively (Figure 3A). From these results we calculated that individuals who carried two copies of the PD ‘protective’ Rep1-259 bp, had reduced levels of human *SNCA*-mRNA, amounting to a nearly 40% decrease relative to individuals that carried at least one copy of the PD-risk associated alleles. Similarly, in the midbrain including SN samples (n = 34) a 50–65% decrease in the average expression level of *SNCA*-mRNA was observed with the 259/259 genotype (n = 3) when compared to each of the other Rep1 genotypes (Figure 3B). This reduction effect of the 259/259 genotype did not show, however, a trend towards significance which might be explained by the small size of the group and the large variability in *SNCA*-mRNA levels within each genotype group (resulting from the neuronal cell heterogeneity of the mid brain/substantia nigra tissue)[12]. In contrast, no significant correlation of the Rep1 site with SNCA-mRNA levels was identified in the frontal cortex (n = 117; P = 0.91), suggesting that Rep1 might affect SNCA expression in a brain-region specific manner (Figure 3C). A summary of the results is listed in Table 4.

**Table 3:** SNPs analyzed in the study with allele frequencies.

SNP	Total No./MAF	Caucasians No./MAF	CEPH† No./MAF
***rs2583988***	69/0.241	63/0.272	102/0.255
***rs2619363***	72/0.25	64/0.274	112/0.24
***rs17016074***	9/0.031	4/0.017	112/0
***rs365165***	128/0.448	98/0.419	0.37*
***rs356219***	120/0.417	88/0.376	112/0.411

No.- total number of alleles (2 per individual); MAF- the minor allele frequency.

†HapMap data base. *MAF reported in PD-SNCA association studies.

**Table 4:** Summary of the genetic correlations between genetic variants at SNCA locus and SNCA-mRNA levels.

		FC	TC	SN
**5′region**	**Rep1**	=	259/259<259/261, 261/261, 259/263, 261/263, 263/263	*259/259<259/261, 261/261, 259/263, 261/263, 263/263
	***rs2583988***	=	=	=
	***rs2619363***	=	=	=
**3′region**	***rs17016074***	=	*AA<GA,GG	*AG<GG
	***rs365165***	=	AA>GA, GG	AA,GA>GG
	***rs356219***	=	AA>GA, GG	AA,GA>GG

‘ = ’ no correlation, *no trend towards significance, FC- frontal cortex, TC-temporal cortex, SN-substantia nigra.

### Effect of SNPs in the 3′ region of *SNCA* on *SNCA*-mRNA levels in different brain tissues

**Figure 4:**
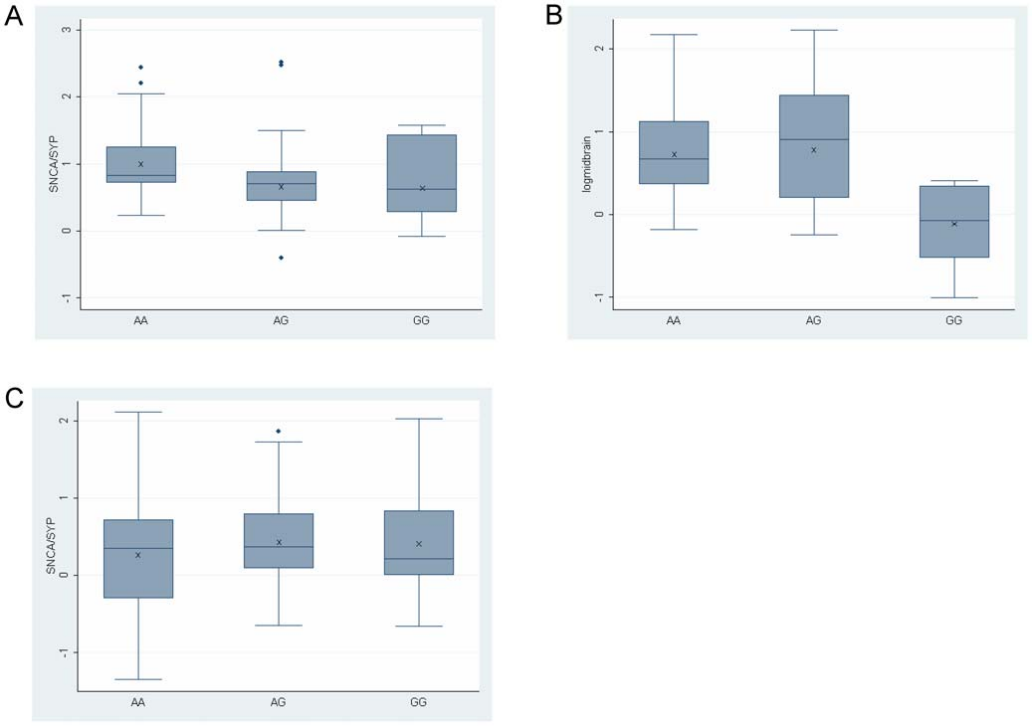
Effect of SNP rs356219, 3′region genotypes, on human *SNCA*-mRNA expression levels in human brains. Individuals were genotyped for SNP rs356219. Three brain regions were analyzed: temporal cortex (A), midbrain including SN (B) and frontal cortex (C). In each brain region fold levels of human *SNCA*-mRNA were assayed by real-time RT-PCR using TaqMan technology and calculated relative to human *SYP*-mRNA reference control using the 2^-ΔCt^ method. (A) Analysis of the temporal cortex showed that the protective genotype AA correlates with higher *SNCA*-mRNA levels than the GA and GG genotypes (P = 0.013). The association trend was confirmed in a subset of samples using also the *GAPDH* and *ENO2* reference genes (Table S2). (B) In the midbrain including SN the AA and AG genotypes correlate with higher SNCA-mRNA levels compared with the GG risk genotype (P<0.05). (C) No correlations of SNP rs356219 genotypes with *SNCA*-mRNA levels were detected in the frontal cortex. For each genotype the box plot represents the analysis performed using all brain samples available from the specific brain region, each of which was analyzed twice independently, each time in duplicate. The average values are presented in ‘X’. The box plot shows the median (horizontal line inside the box) and the 25^th^ and 75^th^ percentiles (horizontal borders of the box). The range between the 25th and 75th percentiles is the interquartile-range (IQR). The whiskers show the minimal and maximal values inside the main data body.

**Figure 5:**
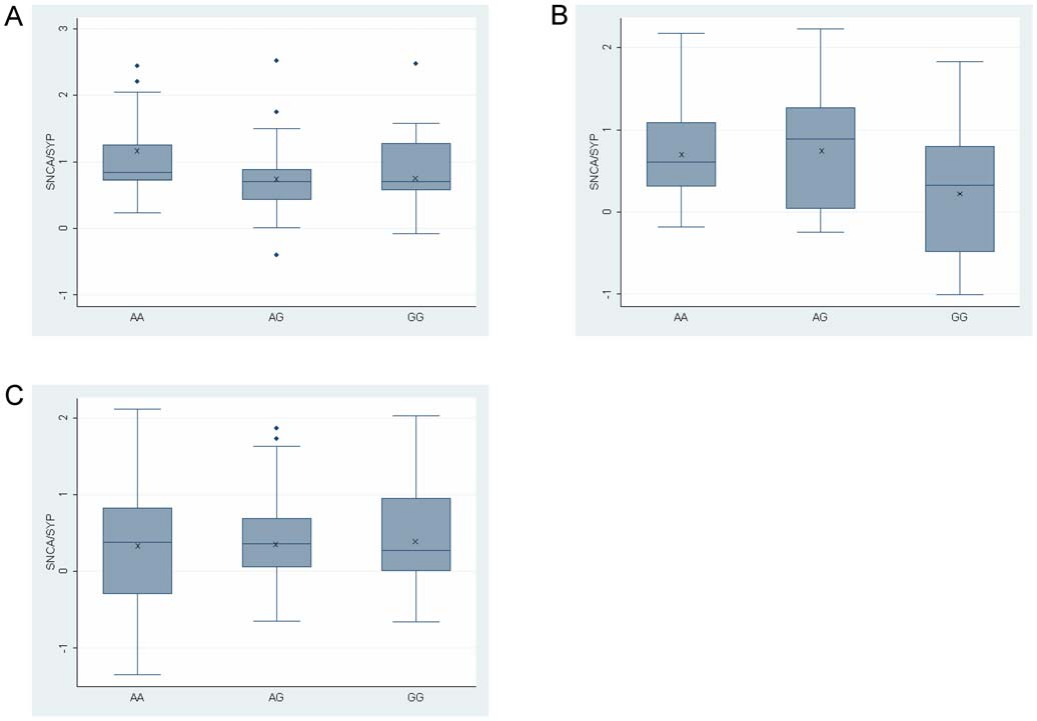
Effect of SNP rs365165, 3′region genotypes, on human *SNCA*-mRNA expression levels in human brains. Individuals were genotyped for SNP rs365165. Three brain regions were analyzed: temporal cortex (A), midbrain including SN (B) and frontal cortex (C). In each brain region fold levels of human *SNCA*-mRNA were assayed by real-time RT-PCR using TaqMan technology and calculated relative to human *SYP*-mRNA reference control using the 2^-ΔΔCt^ method. (A) Analysis of the temporal cortex showed that the protective genotype AA correlates with higher *SNCA*-mRNA levels than the GA and GG genotypes (P<0.05). (B) In the midbrain including SN, the AA and AG genotypes correlate with higher SNCA-mRNA levels compared with the GG risk genotype (P<0.05). (C) No correlations of SNP rs365165 genotypes with SNCA-mRNA levels were detected in the frontal cortex. For each genotype, the box plot represents the analysis performed using all brain samples available from the specific brain region, each of which was analyzed twice independently, each time in duplicate. The average values are presented in ‘X’. The box plot shows the median (horizontal line inside the box) and the 25^th^ and 75^th^ percentiles (horizontal borders of the box). The range between the 25th and 75th percentiles is the interquartile-range (IQR). The whiskers show the minimal and maximal values inside the main data body.

The effect of the *SNCA* 3′ region on *SNCA* mRNA levels was tested with three SNPs: the rare rs17016074 and the two 3′ region common tagging SNPs rs356219 and rs365165 (Figure 1). All samples contained the rs356219, rs365165, and rs17016074 SNP genotypes and the allele frequencies in our study group are summarized in Table 2. Genotypes of SNP rs356219 (G/A) showed an effect on *SNCA* mRNA levels in temporal cortex (P = 0.013) and midbrain including SN tissue (P<0.05) (Figure 4 A and B). In the temporal cortex the homozygous rs356219 ‘protective’ AA genotype (n = 29) showed higher expression levels of SNCA mRNA than the GA and the GG genotypes (n = 38, 10), amounting to a nearly 40% increase (Figure 4A). In the substania nigra, the homozygous AA and the heterozygous GA genotypes (n = 12, 16) correlate with higher *SNCA* mRNA levels than the risk genotype GG (n = 6) (Figure 4B). In the frontal cortex, on the contrary, no correlation was identified between *SNCA* mRNA levels and rs356219 genotypes (Figure 4C). *SNCA* mRNA levels were also correlated with SNP rs365165 (G/A) in temporal cortex (Figure 5A, P<0.05) and midbrain including SN (Figure 5B, P<0.05), following the same correlations of genotypes to *SNCA* mRNA fold expression (Table 4); while no correlation was observed in the frontal cortex (Figure 5C), similar to the results obtained for the downstream *SNCA* 3′ SNP. Thus, as expected based on the high LD between these SNPs, the results observed for SNP rs365165 supported the findings of SNP rs356219 (Table 4). Of note the magnitude of the SNPs effect on SNCA-mRNA fold expression was larger for SNP rs356219. Analysis of the rare (<5%) rs17016074 suggested a possible effect of the minor allele on *SNCA* mRNA reduction, since the homozygous AA (n = 1 of 77) had a lower level of *SNCA* mRNA in the temporal cortex and the heterozygous AG (n = 1 of 34) revealed a lower level in the midbrain including SN, which will need to be explored with a larger group (data not shown). The frontal cortex region did not show a significant correlation with *SNCA* mRNA folds levels with any of the genotypes at the 3′ region SNPs. A summary of the results is listed in Table 4.

## Discussion

Finding interesting correlations between genetic variants and gene expression levels does not necessarily require a comparison between tissues from both affected cases and controls. Significant differences in gene expression levels were also shown to be associated with different genotypes in human tissues of unaffected individuals [24], [25]. Identification of such genetic expression effects in a disease relevant tissue could provide important information for determining which variants to pursue in functional studies and which will further our understanding of the underlying biology of associations with the disease of interest. We previously reported differences in *SNCA*-mRNA expression levels between PD cases and controls[12]. However, in this current report, we analyzed unaffected brains, which allow us to overcome methodological and interpretative challenges that arise from the massive cell loss, particularly neuronal loss, along with other pathologic processes accompanying neurodegeneration that may influence expression. Following this approach, we looked for variations in *SNCA* expression in the brains of unaffected people (age matched to late-onset PD). Specifically, in the present study we focus on genetic regulation of RNA. Therefore, we looked for variations in *SNCA* mRNA levels and tested for association with PD-associated variants positioned within putative regulatory regions for RNA expression: 1) the 5′ region of the gene which presumably influences transcription and 2) the 3′ UTR and 3′ of the gene that most likely affects post-transcriptional regulation. All variants chosen for the present study had been repeatedly reported to confer increased risk for developing PD [14]–[18]. The five analyzed SNPs are located within evolutionary highly conserved regions, and the two 5′ SNPs were also in or very close to potential binding sites for transcription factors (data not shown).

In the 5′ region we found that, among the polymorphic loci tested, only variation at the Rep1 locus was responsible for differences seen in SNCA-mRNA levels. Previously, we extensively characterized the functional significance of the PD-associated Rep1 polymorphic site [26]–[29] and its contribution to the transcriptional regulation of *SNCA* in an *in vitro* cell-based system and *in vivo* using a transgenic mouse model [21], [22], [30]. Recently, Fuchs and colleagues reported Rep1's effect on SNCA protein levels in human blood, but failed to detect an effect in brain samples (N _control subject_ = 24) [31]. Here using a much large sample size (N _control subject_ = 144) we demonstrate for the first time the regulatory effect of Rep1 alleles *in vivo* in human brain structures relevant to the disease providing further direct, functional evidence for the reported genetics associations; i.e. in PD-affected brain regions the ‘protective’ genotype correlated with lower *SNCA*-mRNA levels compared with all the other genotypes carrying one or two PD-risk alleles (261 and 263) [18], [26]–[29]. The direction of the Rep1 alleles' effect on SNCA-mRNA levels is consistent with our previous observations using both a cell-based reporter system[22] and a mouse model [23]. Furthermore, our finding lends support to the general hypothesis that an increase in the expression of *SNCA* may also contribute to the common, ‘idiopathic’ PD phenotype, while decreased *SNCA* levels protect from this devastating disease.

In the 3′ region, we found evidence for a regulatory role for all tested SNPs. In contrast to the Rep1 effect, however, the ‘protective’ genotype at the 3′ region defined by each of the common tagging SNPs is associated with higher *SNCA*-mRNA levels in disease affected brain tissues (midbrain including SN and temporal cortex). With both of the common 3′ region tagging SNPs, rs356219 and rs365165, the ‘protective’ AA genotype correlated with higher *SNCA*-mRNA in the temporal cortex, while both the homozygous ‘protective’ and the heterozygous genotypes (AA and GA) correlated with higher mRNA levels in the midbrain including SN. In general, our findings are in agreement with a recent smaller scale study reporting that the protective rs356219 genotype (AA) is accompanied by higher mRNA levels in the cerebellum, whereas the heterozygous genotype (GA) correlated with the higher mRNA levels in the midbrain including SN. The subtle differences from this previous study might be from the result of the smaller sample size studied [31]. Although these findings run contrary to the conventional hypothesis that lower *SNCA* expression confers PD-protection, it might be that rs356219, rs365165, or any other SNP in the 3′ LD block (extended to include intron 4), exerts a regulatory effect not simply by changing total *SNCA*-mRNA levels but by a different molecular mechanism, such as splicing, to change the relative levels of the different splice forms (e.g., NACP140/112). For example, it was suggested that exon 5 deletion (NACP112) result in enhanced aggregation due to a significant shortening of the unstructured C-terminus[32], [33]. Thus, one can speculate that although the protective genotype led to an increase in the overall *SNCA*-mRNA levels, the proportion of the aggregated isoform may decrease providing PD-protection. Additional assays directly measuring the association of the full and spliced forms with SNPs in the 3′ region will be required to settle this question. Alternatively, acknowledging the contradictory findings in the field with respect to ups and down regulation of *SNCA* levels in PD brains [12], [13], [34]–[36] one cannot exclude the interpretation that the association between the 3′ SNPs and sporadic PD is the result of lower *SNCA* expression levels. Nevertheless, alteration in *SNCA*-mRNA levels might contribute to disease pathogenesis in many but, perhaps, not all cases of sporadic PD.

Our study examined several SNPs in various brain tissues for association with expression, and as such, is subject to false positive associations. Therefore, *P* values should be interpreted with caution. After Bonferroni adjustment for 18 hypotheses (6 variants x 3 tissues), the associations with all variants became non-significant. However, given that these were not independent hypotheses (brain tissues from the same individuals and with clear correlation of expression, SNPs in linkage disequilibrium), this may be an overly conservative interpretation. The results of this study suggest an association between specific variants in *SNCA* and expression that warrants further investigation in a larger cohort such as a multi site meta analysis platform.

Our analysis included functionally and anatomically distinct brain regions from unaffected age-matched brains. Frontal cortex, temporal cortex, and substantia nigra are known to have differential susceptibility to PD pathology, ranging from severely afflicted substantia nigra, followed by temporal cortex that is involved in a later stage, to the frontal cortex, which is a late-developing structure that might be spared PD features [37]. Interestingly the genetic control identified in this study was not global (across all brain regions) but brain-region specific, indicating regionally differential regulation of *SNCA*-mRNA expression. Our results show that, in relation to *SNCA*-mRNA overall expression levels and genetic regulation, the temporal cortex mirrors the substantia nigra. Thus, it is possible that these two brain regions share regulatory mechanisms controlling *SNCA* expression. In contrast, the frontal cortex showed lower SNCA-mRNA levels and did not reflect the genetic regulation observed for the temporal cortex and the midbrain including SN. Thus, concerning the limited availability of substantia nigra region, temporal cortex may serve as a surrogate brain region carry out further studies on expression of *SNCA* and other genes in PD.

A critical question concerning the molecular pathogenesis of PD is what role *SNCA* plays in sporadic PD. Several recent association studies have demonstrated that genetic variability across the *SNCA* locus is associated with susceptibility to sporadic PD in many populations [14]–[17]. In this study, we demonstrated the functional consequence of genetic variations in the *SNCA* genomic region and showed that the genetic association of some variants correlates with biological function, in particular regulation of *SNCA* expression levels. This suggests that regulation of *SNCA* gene expression levels might be important in the development of sporadic PD in patients who do not express a mutated protein or who do not have an increase in gene copy number. Given that multiplications of *SNCA* have been implicated in familial PD, we suggest that a subtle increase in SNCA expression over decades confers an elevated risk for late-onset, sporadic PD.

The results of our study advanced our understanding of the contribution of genetic variants within the *SNCA* locus to sporadic PD. Better understanding the molecular mechanisms modulating *SNCA* gene expression, may lead to novel therapeutic approaches based on reductions in *SNCA* levels [38]–[40].

## Materials and Methods

### Brain Samples

Brain tissue samples, including midbrain/pons/substantia nigra (n = 34), temporal cortex (n = 77), and frontal cortex (n = 117), from neurologically healthy controls (n = 144) (Table 1) were obtained through the Kathleen Price Bryan Brain Bank (KPBBB) at Duke University, the Brain and Tissue Bank for developmental Disorders at the University of Maryland, the Layton Aging & Alzheimer's Disease Center at Oregon Health and Science University, and the National NeuroAIDS Tissue Consortium (NNTC). All post mortem interval (PMI) were <24 hours. Demographics for these samples are included in Table 1. All brain samples were collected from clinically and neuropathologically healthy cadavers who had no evidence of PD, AD or other neurodegenerative disorder at *post mortem* examination.

### DNA Extraction and Genotyping

Genomic DNA was extracted from brain tissues by the standard Qiagen protocol. Genotype determination of each Single Nucleotide Polymorphism (SNP) was performed by allelic discrimination using TaqMan SNP Genotyping Assays (Applied Biosystems, Foster City, CA). Each genomic DNA sample (20 ng) was amplified using TaqMan Universal PCR master mix reagent (Applied Biosystems, Foster City, CA) combined with the specific TaqMan SNP genotyping assay mix corresponding to the genotyped SNP (Table S1). The assays were carried out using the ABI 7900HT and the following conditions: 2 min at 50°C, 10 min at 95°C, 40 cycles: 15 sec at 95°C, and 1 min at 60°C. Genotype determination was performed automatically using the SDS version 2.2 Enterprise Edition Software Suite (Applied Biosystems, Foster City, CA). SNCA-Rep1 dinucleotide complex repeat polymorphism genotyping was carried out by size using previously published method [22], [26], [41]. Briefly: the SNCA-Rep1 region of each genomic DNA sample (20 ng) was PCR-amplified using fluorescently labeled forward FAM 5′-CCTGGCATATTTGATTGCAA-3′ and reverse 5′-GACTGGCCCAAGATTAACCA-3′ primers [19]. Genotypes were determined on an ABI 3730 using GeneMapper version 4.0 software (Applied Biosystems, Foster City, CA) for allelic size assessment. The Rep1 allele was determined according to the length of the PCR product (259 bp, 261 bp, 263 bp and 265 bp). All genotypes were tested for Hardy-Weinberg Equilibrium and allele frequencies compared to the public database and previously published results (Tables 2 and 3).

### RNA extraction and cDNA synthesis

Total RNA was extracted from brain samples (100 mg) using TRIzol reagent (Invitrogen, Carlsbad, CA) followed by purification with an RNeasy kit (Qiagen, Valencia, CA) following the manufacturer's protocol. RNA concentration was determined spectrophotometrically at 260 nm, while the quality of the purification was determined by 260 nm/280 nm ratio that showed values between 1.9 and 2.1, indicating high RNA quality. Additionally, quality of sample and lack of significant degradation products was confirmed on an Agilent Bioanalyzer. The RNA Integrity Number (RIN) measurements were greater than 7 validating the RNA quality control. Next, cDNA was synthesized using MultiScribe RT enzyme (Applied Biosystems, Foster City, CA) under the following conditions: 10 min at 25°C and 120 min at 37°C.

### Real time PCR

Real-time PCR was used to quantify human *SNCA* mRNA levels as previously described [12]. Briefly, duplicates of each sample were assayed by relative quantitative real-time PCR using the ABI 7900 for analysis of the level of *SNCA* message as compared in brain tissues to mRNA encoding human synaptophysin (*SYP),* a presynaptic protein that has a similar expression pattern to *SNCA* [12], [30]. Each cDNA (10 ng) was amplified in duplicate in at least two independent runs (overall≥4 repeats), using TaqMan Universal PCR master mix reagent (Applied Biosystems, Foster City, CA) and the following conditions: 2 min at 50°C, 10 min at 95°C, 40 cycles: 15 sec at 95°C, and 1 min at 60°C. The target *SNCA* cDNA was amplified using ABI MGB probe and primer set assay ID Hs00240906_m1, normalized to a *SYP* RNA control (ABI MGB probe and primer set assay ID Hs00300531_m1) (Applied Biosystems, Foster City, CA). As a negative control for the specificity of the amplification and to control for DNA contamination, we used RNA control samples that were not converted to cDNA (no-RT) and no-cDNA/RNA samples (no-template) in each plate. No observable amplification was detected. Data were analyzed with a threshold set in the linear range of amplification. The cycle number at which any particular sample crossed that threshold (Ct) was then used to determine fold difference. Fold difference was calculated as 2^−ΔΔCt^; ΔCt = [Ct(*SNCA*)-Ct (*SYP*)]. ΔΔCt = [ΔCt(sample)]-[ ΔCt(calibrator)]. The calibrator was a particular brain RNA sample used repeatedly in each plate for normalization within and across runs. The variation of the ΔCt values among the calibrator replicates was smaller than 10%.

Of note is that three internal controls were compared: the neuronal specific genes *Enolase 2* (*ENO2* Hs00157360_m1) and synaptophysin (*SYP* Hs00300531_m1) and the house keeping gene glyceraldehydes-3-phosphate dehydrogenase (*GAPDH* Hs00999905_m1). For assay validation we generated standard curves for *SNCA* and each reference assay, *ENO2, SYP* and *GAPDH* using different amounts of human brain total RNA (0.1–100 ng). The slope of the relative efficiency plot for *SNCA* and each internal control (*ENO2 SYP* or *GAPDH*) was determined to validate the assays. The slope in the relative efficiency plots for *SNCA* and the reference genes were >0.1, showing a standard value required for the validation of the relative quantitative method (Figure S1). In addition, for a subset of brain samples (for each type of brain tissue) we used the geometric mean of *SYP* and *ENO2* and the *GAPDH* as normalization controls and confirmed the selection of *SYP* as a representative normalization control for the entire brain set. Thus, for the extended study we chose *SYP* as the internal control.

### Statistical analysis

*SNCA*-mRNA fold expression value of each sample was analyzed repetitively and the results of all replicates were averaged. All average values were expressed as mean±S.E.M. Correlations were assessed by linear regression analyses. A log transformation (log2) was performed on all mRNA levels to assure normal distribution [42]. The general linear model (GLM) method was used to evaluate the effect of the primary explained variable (genotype) as well as other secondary variables (sex, age, PMI, ethnicity, tissue source) on the RNA levels. The GLM is a procedure unifying the ordinary linear regression and ANOVA as well as other procedures based on the least square computation such as ANCOVA. Since gender, age, PMI, ethnicity, and tissue source may also show an effect on the RNA levels, they were included in the model as factors. Where the *P* value of the maximal model remains significant, an effect of each single term was estimated calculating the type III sum of squares and the corresponding *F* value and its probability *P*. Correction for multiple testing employed the Bonferroni method. Tissues comparisons were done by paired t-tests. All analyses were carried out using STATA/IC10.0 statistical software (StataCorp, College Station, TX).

The Bryan ADRC Autopsy and Brain Donation Program Database/Repository has been granted approval from the Duke University Health System Institutional Review Board for Clinical Investigations, eIRB# Pro 00016278. Subject's (or their Legally Authorized Representatives) participating in the Bryan ADRC Autopsy and Brain Donation Program Database/Repository have provided written consent for use of their data and brain specimens to be used for use in future research. The genetic and expression analysis of all brain tissues obtained for this study is covered by eIRB exemption #10141.

## Supporting Information

Table S1 TAQMAN Genotyping Assays *Primers and probe sequences available upon request.
(0.03 MB DOC)

Table S2 Discovery and Confirmatory Samples Sets. FC- frontal cortex, TC-temporal cortex, SN-substantia nigra. PMI- post mortem interval. Total no.- indicates the entire samples set used in the initial discovery step (N = 228; reference gene, SYP); Replication- referrers to the subset of temporal cortex samples used in the validation step (n = 24; reference genes, SYP, ENO2 and GAPDH) to confirm key results.
(0.03 MB DOC)

Figure S1 Relative efficiency plots of SNCA and the reference control genes. Validation curve of the Δreal time assay for relative quantization of human SNCA-mRNA in brain relative to: (A) SYP-mRNA, (B) ENO2-mRNA, and (C) GAPDH-mRNA. Relative efficiency plots of SNCA and each of the normalization control genes were formed by plotting the log input amount (ng of total RNA) versus the ΔCt = [Ct(SNCA)-Ct(SYP/ENO2/GAPDH)]. The slopes are all <0.1, which indicated the validation of the ΔCt calculation in the range between 0.1–100 ng RNA with all three controls.
(1.94 MB TIF)
