# Supplementary material for: Genetic Regulation of α-Synuclein mRNA Expression in Various Human Brain Tissues
Source: PLoS One. 2009 Oct 16;4(10):e7480. doi: 10.1371/journal.pone.0007480 (PMC2759540; doi:10.1371/journal.pone.0007480)
Supplement: Table S1 — TAQMAN Genotyping Assays *Primers and probe sequences available upon request. (0.03 MB DOC) [file pone.0007480.s001.doc]

| **SNP** | **Assay #** |
| --- | --- |
| rs356219 | C__1020193_10 |
| rs2583988 | C__16258378_10 |
| rs2619363 | C__16036895_10 |
| rs17016074 | C__32615669_10 |
| rs356165 | Custom* |
