# Supplementary material for: Genetic Regulation of α-Synuclein mRNA Expression in Various Human Brain Tissues
Source: PLoS One. 2009 Oct 16;4(10):e7480. doi: 10.1371/journal.pone.0007480 (PMC2759540; doi:10.1371/journal.pone.0007480)
Supplement: Table S2 — Discovery and Confirmatory Samples Sets. FC- frontal cortex, TC-temporal cortex, SN-substantia nigra. PMI- post mortem interval. Total no.- indicates the entire samples set used in the initial discovery step (N = 228; reference gene, SYP); Replication- referrers to the subset of temporal cortex samples used in the validation step (n = 24; reference genes, SYP, ENO2 and GAPDH) to confirm key results. (0.03 MB DOC) [file pone.0007480.s002.doc]

|  | **All subjects** | **FC** | **TC** | **SN** | **FC+TC+SN** | **FC+TC** | | **FC+SN** |
| --- | --- | --- | --- | --- | --- | --- | --- | --- |
| **Total no.** | 144 | 117 | 77 | 34 | 7 | 68 | 2 | |
| **Replication** | 24 |  | 24 |  | 7 | 17 |  | |
