# Supplementary figures and images for: Genetic Regulation of α-Synuclein mRNA Expression in Various Human Brain Tissues
Source: PLoS One. 2009 Oct 16;4(10):e7480. doi: 10.1371/journal.pone.0007480 (PMC2759540; doi:10.1371/journal.pone.0007480)

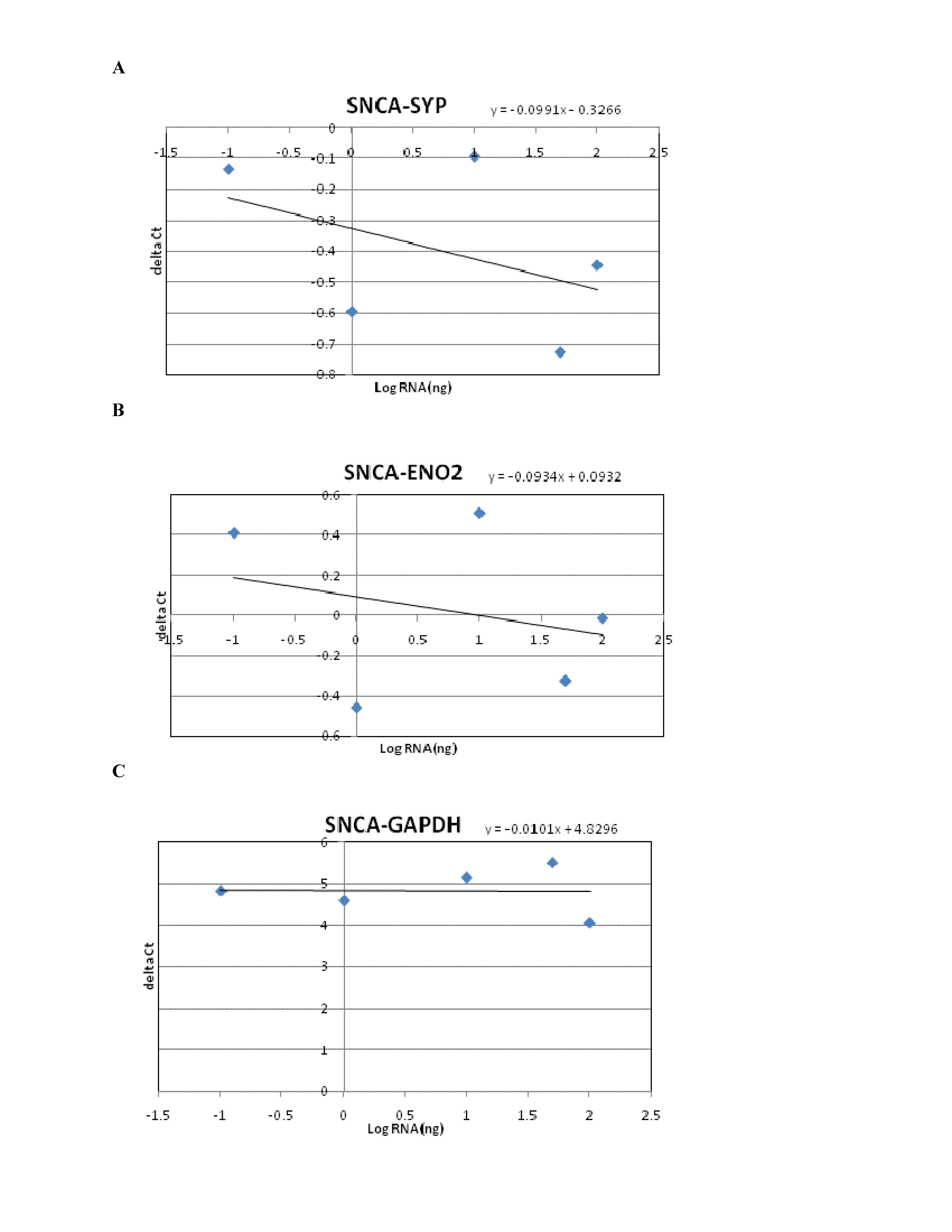

Supplement: Figure S1 — Relative efficiency plots of SNCA and the reference control genes. Validation curve of the Δreal time assay for relative quantization of human SNCA-mRNA in brain relative to: (A) SYP-mRNA, (B) ENO2-mRNA, and (C) GAPDH-mRNA. Relative efficiency plots of SNCA and each of the normalization control genes were formed by plotting the log input amount (ng of total RNA) versus the ΔCt = [Ct(SNCA)-Ct(SYP/ENO2/GAPDH)]. The slopes are all <0.1, which indicated the validation of the ΔCt calculation in the range between 0.1–100 ng RNA with all three controls. (1.94 MB TIF) [file pone.0007480.s003.tif]
